# Supplementary Feeding of Grazing Inner Mongolian Cashmere Goats during Pregnancy—Based on “Nutrient Requirements of Cashmere Goats”

**DOI:** 10.3390/ani13030473

**Published:** 2023-01-29

**Authors:** Xin-Hui Wang, Qing Li, Zi-Bin Zheng, Xiao-Gao Diao, Li-Wen He, Wei Zhang

**Affiliations:** State Key Laboratory of Animal Nutrition, College of Animal Science and Technology, China Agricultural University, Beijing 100193, China

**Keywords:** cashmere goats, nutrition and management, gestation, growth performance, fiber quality, hair follicle development

## Abstract

**Simple Summary:**

In the fall and winter, nutrient supplies of forage cannot meet the needs of pregnant cashmere goats, and there lacks clear precise supplemental feeding for them under grazing. This study first applied “Nutrient Requirements of Cashmere Goats” to supplementary feeding for pregnant Inner Mongolian cashmere goats under grazing. Supplementation increased pregnant goat cashmere length, cashmere yield, body weight after shearing, single born kid weight, twin-birth kid weight and kids’ mature secondary hair follicle density. The results demonstrated that the supplementary feeding based on the standard could enhance pregnant goats’ production performance.

**Abstract:**

This study aimed to conduct precise supplementation for pregnant cashmere goats under grazing based on the feeding standard. Eight Inner Mongolian pregnant cashmere goats of near-average body weight were selected at early gestation (44.41 ± 4.03 kg) and late gestation (46.54 ± 4.02 kg) to measure their nutrient intake. Then, two pregnant cashmere goat flocks, No. 10 (control group, on-farm supplement) and No. 11 (supplemented group, supplement based on standard), with the same goat herd structure and grassland type, were chosen to conduct the supplemental feeding experiment. The results showed that pregnant cashmere goats lacked daily the intake of dry matter, digestive energy, crude protein and most essential mineral elements under grazing. After supplemental feeding, the supplementation based on the feeding standard increased the cashmere length and cashmere length growth volume and decreased the cashmere fineness, with no statistical significance. The goat cashmere yield, goat weight after shearing, single and twin-birth kid weight and kids’ mature secondary hair follicle density were significantly higher in the supplemented group (*p* < 0.05). In conclusion, supplementation in accordance with “Nutrient Requirements of Cashmere Goats” can enhance pregnant cashmere goats’ fiber production, growth performance, fertility and kids’ secondary hair follicles development, which is of great importance for the healthy and precise nutrition and management of cashmere goats.

## 1. Introduction

Cashmere goats are mainly distributed in Central Asia and Mongolia, especially in Western China [1]. Inner Mongolia cashmere goats are known worldwide for their high-quality cashmere fiber; moreover, they are also raised locally for meat and milk products [2]. In traditional management, Inner Mongolia cashmere goats usually graze on a full grazing system with only forage as feed. It has been reported that the crude protein (CP) content of nature grasses decreases from 9.57%–21.26% to 2.58%–10.03% and neutral washing fiber (NDF) and acid detergent fiber (ADF) contents increase gradually from summer to the winter in the YiWei White Cashmere Goat Farm located in the Inner Mongolia Autonomous Region [3]. Moreover, the mineral element level in the soil–grazing–livestock ecosystem of the *Stipa breviflora* steppe located in the Inner Mongolia Siziwangqi Region is high in Fe, Mn and Ca, low in P, Na, K and Se, and seasonally deficient in Cu, Zn and S [4,5]. The nutritional value of natural grasslands varies seasonally, and the pasture nutritional value may not meet the needs of pregnant ruminants in the dry and autumn winter season [6,7,8]. It has been shown that the energy or protein deficiency of pregnant sheep or goats can inhibit the development of fetal renal vasculature and renal units, resulting in an imbalance in superoxide dismutase, hydrogen peroxide inactivation systems in the thymus, injury of the jejunum, antioxidant capacity, and immune responses of newborn lambs or kids [9,10,11]. A study has shown that in an extensive farming system, electrolyte levels of female goats display different concentrations in the bloodstream according to physiological stage and kid numbers, and it is necessary to consider these differences in actual feed and management [12]. The mineral element deficiency decreases production performance, resulting in associated illnesses, even death, especially Cu, Zn and S, which are particularly important for wool-bearing animals; their deficiency can affect fur color and traits; wool yield, elasticity and strength; and animal reproductive performance, etc. [13,14].

Not only the Inner Mongolia region, but also many other parts of the world are faced with low pasture nutritional value and limits for grazing animal growth. In the natural grassland of Dangxiong, Tibet, the nutritional value of forage grasses during the spring re-greening and summer vigorous periods is significantly higher than that during the autumn and winter dry periods, and the intake of Ca and P by grazing pregnant ewes is insufficient in alpine meadows during the winter dry period and in river meadows during the summer peak period [15]. In Ethiopia and the semi-arid regions of northeastern Brazil, the quantity and quality of natural pasture resources limit the production of grazing goats, and supplemental feeding is often used as an effective measure to improve the growth performance and production performance of goats [16,17]. Hence, supplemental feeding is necessary for grazing pregnant goats in the dry and autumn winter season [18].

However, before the promulgation of “Nutrient Requirements of Cashmere Goats”, supplemental feeding in actual production was mainly based on NRC (1981), AFRC (1998) and experience, which were not suitable for the actual production of cashmere goats [19]. It has been reported that 300 g/day of corn is the only feedstuff used as supplementary feeding for castrated goat kids and female kids in the YiWei White Cashmere Goat Farm located in the main producing area of Inner Mongolia cashmere goats [3]. Similarly, McGregor et al. provided 300 g/day grain supplements for cashmere goats, which significantly increased the total cashmere diameter (2.12 µm) and made cashmere lose its economic value [20]. It remains unclear how to supplement feeding pregnant cashmere goats scientifically and precisely under pasture with a poor nutrient content. “Nutrient Requirements of Cashmere Goats (NY/T4048–2021)”, China’s and the world’s first cashmere goat feeding standards, was published in 2021 [21], which provides a nutrition reference for the accurate supplementary feeding of pregnant cashmere goats. Hence, in this study, “Nutrient Requirements of Cashmere Goats” was used as a guide to provide supplement diets for goats during early and late gestation. Comparing relevant indicators with on-farm supplementation, the aim of this study was to investigate the effectiveness of supplementary feeding based on the feeding standard for Inner Mongolian Cashmere Goats during pregnancy and provide a theoretical and data basis for the precise feeding of cashmere goats in other physiological stages. This will give full play to the advantages of germplasm resources of cashmere goats and guide and promote the high-quality and sustainable development of the cashmere goat industry.

## 2. Materials and Methods

### 2.1. Animals and Experimental Procedure

The experiment was conducted at a commercial farm (YiWei White Cashmere Goat Farm) located in the Inner Mongolia Autonomous Region (39°11′ N, 107°16′ E), which is the largest national alba cashmere goat breeding farm and national alba cashmere goat standardized breeding demonstration zone. In the experiment area during fall and winter, *Stipa breviflora Griseb*, *Peganum harmala L* and *Oxytropis aciphylla Ledeb* were the dominant plant species in natural pastures.

First, eight Inner Mongolian alba cashmere goats close to an average body weight were selected at early gestation (44.41 ± 4.03 kg, gestation 60 days) and late gestation (46.54 ± 4.02 kg, gestation 90 days). Moreover, the saturated alkanes were used as indicators to determine the forage nutrition intake [22,23]. Every experiment period was ten days. All goats were given one C_32_ n-alkane capsule (48.5 mg, TARU, early gestation; 37.5 mg, CAU, late gestation) every morning before grazing. Moreover, 0.3 kg DM corn per goat was supplied after grazing at early gestation, with no supplementary feed at late gestation.

Then, two goat flocks, No. 10 (control group, on-farm supplementary feeding management) and No. 11 (supplemented group, supplementation based on standard, on-farm management), with the same goat herd structure and grassland type, were chosen to conduct the supplemental feeding experiment at early and late gestation. The composition and nutritional levels of the supplementation for the two groups are shown in Table 1 and Table 2. Goats in the two experiments above were herded from 8:00 to 18:00.

### 2.2. Sample Collection and Chemical Analyses

In the forage dry matter intake (DMI) determination experiment, every kind of forage sample was collected from day 1 to day 5. Fecal samples were collected before and after grazing from day 6 to day 10 [24]. In addition, the supplemental corn sample was collected in early gestation. The concentrations of alkanes [25], GE (gross energy), CP, Ca, P, NDF and ADF in feces samples, forage samples and supplemental corn samples were determined [26], the content of other mineral elements (K, Mg, Co, Cu, Fe, Mn, Se, Zn and S) in each kind of eaten forage was determined [27,28].

In the supplemental feeding experiment, fifteen pregnant goats close to an average weight in two groups were selected randomly and respectively, and cashmere was collected before and after the supplemental feeding experiment, and cashmere length and cashmere fineness were determined [2]. After kids were born, production data of the two groups were gathered, their birthweights were measured and the kidding rate, single birth rate and twinning rate were calculated [29]. In April, the cashmere fibers were combed out from skin, and the cashmere yields and body weights of goats were determined after shearing. In the newborn kid, 15-days-old, the skin was sampled and stained, and hair follicles numbers were determined [2].

### 2.3. Calculation

After the forage DMI determination experiment, the forage intake proportion for pregnant goats can be calculated based on feces and forage C_27_–C_31_ odd-chain alkane concentrations [22], and the recoveries of C_27_–C_31_ chain alkanes were C_27_: 0.47, C_29_: 0.71 and C_31_: 0.69, respectively [30]. Forage DMI for goats at early and late gestation was calculated with Equations (1) and (2), respectively [23]:

DMI (kg DM) = [F_i_/F_j_ (D_j_ + I_c_ × C_j_) − I_c_ × C_i_]/(H_i_ − F_i_/F_j_ × H_j_), (1)

DMI (kg DM) = (F_i_/F_j_ × D_j_)/(H_i_ − F_i_/F_j_ × H_j_), (2)

and the digestive energy (DE) intake of forage for goats at early and late gestation was calculated using Equations (3) and (4), respectively:

DE_Forage_ (MJ DM) = GE_Forage_ + GE_Corn_ − FE − DE_Corn_, (3)

DE_Forage_ (MJ DM) = GE_Forage_ − FE, (4)

During forage DMI determination experiment, goats were supplemented with corn after grazing in early gestation and supplemented with nothing in late gestation; Equations (3) and (4) were derived from DE = GE − FE [31]. Between Equations (3) and (4), for GE_Forage_, the GE level of forage eate, was the sum of the products of each forage eaten ratio, forage DMI (kg) and corresponding GE (MJ/kg); for DE_Corn_, the DE level of supplemental corn was the estimated as DE = 18.653 − 8.751ADF − 6.667NDF − 4.255CP [32]; FE was the products of feces output (kg) and fecal GE (MJ/kg), and the feces output was indirectly estimated from DMI and dry matter digestibility (DMD); DMD was estimated based on C_31_ as an endogenous indicator [24].

Nutrient deficit and surplus are the gap between each nutrient intake of goats and the corresponding feeding standard. Moreover, forage nutrient intake is the sum of the product of each forage intake proportion and the corresponding forage nutrient (CP, Ca, P, K, Mg, Co, Cu, Fe, Mn, Se, Zn and S) content and forage DMI.

### 2.4. Statistical Analysis

The data of this experiment were analyzed via T-test analysis using SPSS 25.0 (IBM, New York, NY, USA), and the significance level was *p* < 0.05.

## 3. Results

### 3.1. Nutritional Surplus and Deficit of Inner Mongolian Goats during Pregnancy under Grazing Conditions

**Figure 1:**
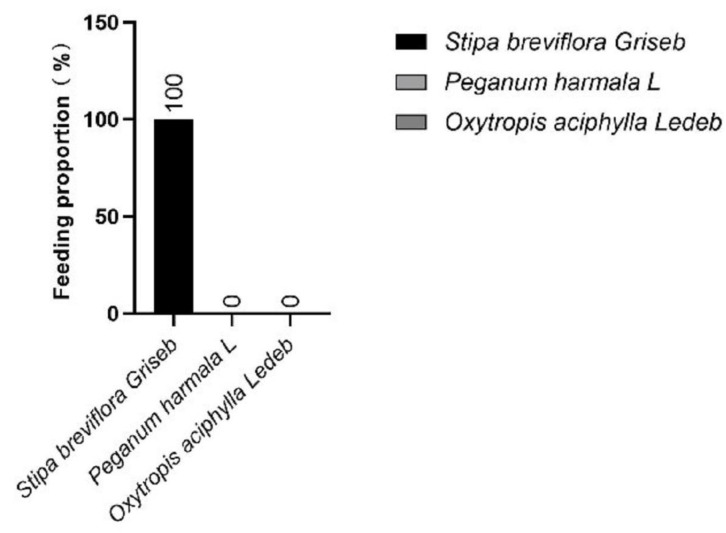
Forage intake proportion of cashmere goats in early gestation (%).

**Figure 2:**
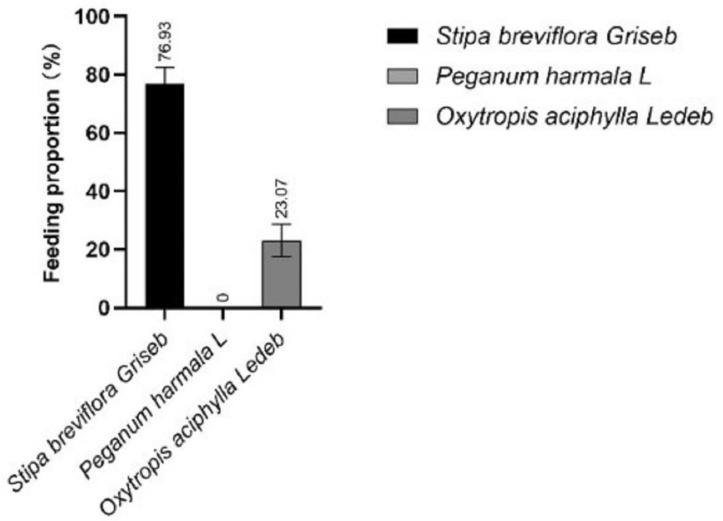
Forage intake proportion of cashmere goats in late gestation (%).

Based on the concentration of alkanes in the feces and forage in early and late gestation (Supplementary Table S1), we first calculated the forage intake proportion. The cashmere goats mainly ate *Stipa breviflora Griseb* in early gestation under grazing (Figure 1), while late-gestation grazing cashmere goats mainly ate *Stipa breviflora Griseb* with small amounts of *Oxytropis aciphylla Ledeb*, and eating ratios were 76.93% and 23.07%, respectively (Figure 2). The nutritional surplus and deficit of goats in early and late gestation were calculated based on the forage intake ratio, forage nutrient content (Supplementary Tables S2 and S3) and forage DMI. Compared with the “Nutrient Requirements of Cashmere Goats”, only the daily intake of Ca, Fe, Mn and Co in early gestation met the goats’ requirements; the goats lacked the daily intake of DMI, DE, CP, P, Cu, Zn, K, Mg, Se and S (Table 3). The calcium-to-phosphorus intake ratio was 7.82:1. Only the daily intake of Fe, Mn and Co in late gestation met the goats’ requirements; the goats lacked the daily intake of DMI, DE, CP, Ca, P, Cu, Zn, K, Mg, Se and S (Table 3). The proportion of calcium and phosphorus intake was 7.29:1.

### 3.2. Supplementation Based on the Standard can Enhance Pregant Cashmere Goat Production Performance

Supplemental feeding for 3 months according to the “Nutrient Requirements of Cashmere Goats” increased pregnant goats’ cashmere length and cashmere length growth volume by 8.50% and 25.89% (*p* > 0.05) and decreased cashmere fineness by 4.12% (*p* > 0.05). In addition, supplementation according to the standard significantly increased cashmere yields in pregnant goats by 7.89% (*p* < 0.01) (Table 4).

### 3.3. Supplementation Based on the Standard can Enhance Pregant Cashmere Goat Growth Performance

Supplemental feeding for 3 months according to the “Nutrient Requirements of Cashmere Goats” could significantly increase the body weights of cashmere goats after shearing (*p* < 0.01), with an increase of 5.71% compared to that in the control group (Table 5).

### 3.4. Supplementation Based on the Standard can Enhance Pregant Cashmere Goat Fertility

After supplemental feeding according to the “Nutrient Requirements of Cashmere Goats”, the kidding rates of the supplemented and control groups were 112.10% and 114.29%, single birth rates were 88.31% and 85.71% and twinning rates were 11.29% and 14.29%, respectively (Table 6)The supplementation based on the standard significantly increased the kid birth weight, single-born kid weight (*p* < 0.01) and twin-birth kid weight (*p* > 0.05) by 3.99%, 4.29% and 0.38% (Table 7), respectively.

### 3.5. Supplementation Based on the Standard can Enhance Hair Follicle Development in Kids

**Figure 3:**
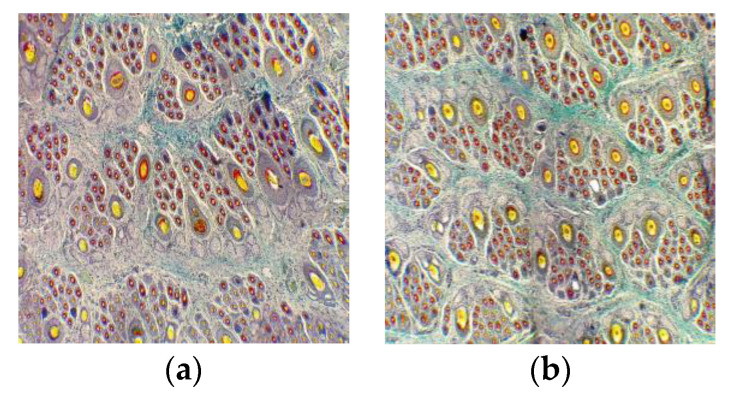
Representative hair follicle groups based on horizontal sections stained via the Sacpic method in control and supplemented groups (40×). (**a**) Representative hair follicle groups in the skin of kids in the control group at the age of 15 days; (**b**) representative hair follicle groups in the skin of kids in the supplemented group at the age of 15 days.

**Figure 4:**
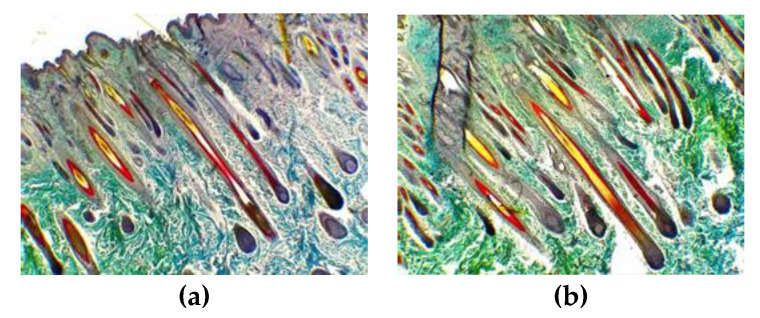
Representative hair follicle groups based on longitudinal sections stained via the Sacpic method in control and supplemented groups (40×). (**a**) Representative hair follicle groups in the skin of kids in the control group at the age of 15 days; (**b**) representative hair follicle groups in the skin of kids in the supplemented group at the age of 15 days.

After the supplemental feeding for 3 months according to the “Nutrient Requirements of Cashmere Goats”, we sampled and stained the skin of newborn kids, and the transverse section showed that more mature secondary hair follicles (SF) were found in the supplemented group than in the control group (Figure 3). The longitudinal section showed that the SFs in the supplemented group were closer to the roots of the primary hair follicles (PF), indicating that the SFs in the supplemented group were in a better state of development than those in the control group (Figure 4).

We compared PF densities, SF densities and S:P values in kids 15-days-old at birth and found no significant differences in PF, SF and S:P values, but the supplementation based on the standard significantly increased the mature SF density (*p* < 0.05) by 12.21% (Table 8).

## 4. Discussion

This study indicated that, compared with that in the “Nutrient Requirements of Cashmere Goats”, the daily intake of DMI, DE, CP, P, Cu, Zn, K, Mg, Se and S of goats in early gestation under grazing conditions was not enough, and the goats in late gestation lacked Ca on this basis. The forage DMI at early gestation, 0.85 kg, in this study was similar to 0.83 kg for Inner Mongolia cashmere goats during the fattening period determined by Wenqi; both studies were carried out using the saturated alkane method [3]. In late gestation, the decrease in forage DMI may be caused by the decrease in forage palatability and increase in the NDF content of the pasture [33]. Similarly, Xinjiang fine-wool sheep has been reported in which intakes of ME and CP were 5.15 MJ/day·ewe and 85.8 g/day·ewe lower than the nutrient requirements of sheep under winter grazing conditions, respectively [34]. As mentioned above, the mineral element level in the soil–grazing–livestock ecosystem of the *Stipa breviflora* steppe located in the Inner Mongolia Siziwangqi Region was high in Fe, Mn and Ca, low in P, Na, K and Se, and seasonally deficient in Cu, Zn and S. Moreover, the result of the mineral element surplus and deficit in this study was similar with that in [4,5], and the results in our study are similar, except that cashmere goats were deficient in Ca intake in late pregnancy. On the one hand, the experimental animals in the two studies were different, and on the other hand, the Ca requirement of cashmere goats became greater into late gestation, and the Ca content in the pasture decreased. In addition, the goats’ intake ratio of Ca and P in this study approached to 7:1, which reduced P absorption, further reducing the absorption of P and Ca [35]. The above results also show that it is necessary to supplement the cashmere goats in this study.

Cashmere or wool yield can be enhanced by supplementing with energy and protein [36,37,38]. Mineral elements are related to the cashmere yield, Zn showed a significant positive correlation with the cashmere percentage [39] and supplementation with 25 mg/kg DM Cu from copper sulfate or copper methionine can enhance cashmere production [40]. Cashmere length and cashmere fineness are relevant to energy, CP and mineral elements, while too much energy makes the cashmere get thicker and lose economic value [41]. Protein has no effect on cashmere fineness when protein levels meet the maintenance needs of goats [42]. Usually, cashmere fibers reached their finest at medium protein and low energy levels [43]. It was reported that supplementation with 20 mg/kg DM (total dietary Cu level of 25.6 mg/kg DM) can promote cashmere growth [44]. However, in this study, the cashmere length increased and the lower cashmere fineness were not statistically significant. A similar result has been reported; under grazing conditions, pregnant Inner Mongolia cashmere goats were supplemented with 9.73 MJ/kg DE + 9.9% CP in the early stage (1st December–1st February) and with 9.46 MJ/kg DE + 9.52% CP in the later period (1st February–31st March), which significantly increased the cashmere yield of goats, but had no statistical effect on cashmere length and cashmere fineness [45]. This is perhaps because the supplementary feeding experiment was performed in a non-increasing period and the secondary hair follicles of Inner Mongolian cashmere goats were in a state of relative ‘rest’ (telogen) during this period (December to March), when the hair shaft gradually stopped growing and shed [46]. These results indicate that cashmere production performance can be enhanced by the supplementary feeding of pregnant cashmere goats according to the feeding standard, and compared with on-farm supplementary, a supplementary feeding scheme is reasonable.

In Liaoning cashmere goats, studies showed that growth traits are closely related to the cashmere yield, and body weight had the greatest direct effect on the cashmere yield [47]. Similarly, Rayeni cashmere goat total fleece weight increased by 45 g and post-weight after shearing was enhanced by 1.5 g after supplementation with a 10.12 MJ/kg diet during pregnancy and lactation periods [48]. It was reported that there is a significant effect of ewe weight on lamb weight [49], and the single and twin-birth kid weight was also increased due to supplementation in this study. Similarly, Ewes’ liveweight gain significantly increased by 134 g/day and lamb birth weights were significantly enhanced by 1 kg, with a supplement of 150 g cottonseed meal + 50 g molasses daily for medium-wooled Peppin Merino ewes during late gestation (last 4 weeks) [50]. Palm kernel cake (PKC) provides a protein source in a supplement (comprising 35% crushed maize, 30% rice bran, 32% PKC, 2% vitamin mineral premix and 1% salt), and supplementation at 0.5% of the live weight significantly enhanced the weight by 0.93 kg after 82 days of the feeding trial for Boer × local female goats (12.4 ± 2.6 kg, 7–9 months) [51]. In addition, supplementation of ewes with Zn, Se and Co in late gestation improved the mineral status of ewes and their kids before weaning and increased lamb weights [52,53]. These results suggested that supplementation based on the standard can enhance pregnant goats’ growth performance and fertility. Studies showed that there is a positive relationship between the lamb birth weight and lamb growth rate [54], and we speculate that supplementation may have a sustained impact on the growth performance of offspring kids, which requires continuous follow-up to verify.

Supplementation based on the standard for pregnant cashmere goats enhanced the mature SF density of kids in this study. Similarly, kids’ SF density can be increased significantly at 12.30 and 28.09 n/mm^2^ with supplements of 5 g urea + 7.5 g Na_2_SO_4_ and 250 g corn in cashmere goats during mid and late gestation, respectively [55]. It was reported that supplementation with Nano-Selenium (declared Se content 0.5 mg/kg DM daily) for cashmere goats during pregnancy can to promote the development and growth of fetal hair follicles [56]. In Angora goats, supplements provided to goats in the middle gestation and lactation stage had a positive effect on the SF density and SF number in kids at all ages after birth, increasing the S:P at 4, 6 and 15 months of age [57]. We speculate that supplementation may improve the hair follicle development of offspring lambs for a long time in this study, which requires continuous follow-up to verify.

## 5. Conclusions

In conclusion, offering a supplemental diet according to the standard could improve the cashmere production performance, growth and fertility of pregnant cashmere goats and improve secondary hair follicle development in their kids. Thus, we concluded that the supplementary feeding based on the standard could enhance pregnant cashmere goats’ production performance, and the supplementary feeding effect is good. In the future, we can continue to track the growth performance and cashmere performance of the kids in the supplemented group and further explore the sustainable impact of maternal supplementary feeding on the offspring lambs. In addition, we will evaluate the nutrient intake of cashmere goats in other physiological stages under grazing conditions and provide precise supplementary feeding based on the standard.

## Figures and Tables

**Table 1:** Supplementary feed ingredients in early and late gestation.

Item	Early Gestation		Late Gestation	
	Ingredients	Proportion (% DM)	Ingredients	Proportion (% DM)
Control group	Corn	100.00	Corn	50.00
			Concentrate feed	50.00
Supplemented group	Corn	60.76	Corn	41.98
	Soybean meal	14.87	Corn stalk	27.92
	Corn stalk	19.51	Soybean meal	26.59
	CuSO_4_·5H_2_O	0.02	CaCO_3_	0.76
	ZnO	0.02	CuSO_4_·5H_2_O	0.02
	K_2_SO_4_	1.50	ZnSO_4_	0.05
	Na_2_SeO_3_	0.003	K_2_SO_4_	1.21
	NaCl	0.42	KH_2_PO_4_	0.62
	NH_4_H_2_PO_4_	0.57	NH_4_H_2_PO_4_	0.42
	CaSO_4_·2H_2_O	0.67	Na_2_SeO_3_	0.00002
			NaCl	0.36

Note: During the supplementary feeding experiment in early pregnancy (15 December 2021–15 January 2022), the goats in the control group and supplemented group were fed 0.3 kg DM corn and fed 0.549 kg DM supplementation per goat every day after grazing; during the period of supplementary feeding in late pregnancy (15 January 2022–15 March 2022), the goats in the control group were fed 0.275 kg DM supplementary feed per goat every day after grazing in mid-January, which increased by 0.025 kg DM supplementary feed every half month, until mid-March, increased to 0.375 kg, and the goats in the supplemented group were fed 0.714 kg DM supplementation per goat every day after grazing.

**Table 2:** Supplementary feed nutrition level in early and late gestation.

Item	Early Gestation		Late Gestation	
Chemical Composition (DM)	Control Group	Supplemented Group	Control Group	Supplemented Group
DE (MJ/kg)	15.26	12.16	14.48	13.41
CP (g/kg)	77.49	114.13	174.40	164.00
Ca (g/kg)	1.39	1.85	12.82	3.61
P (g/kg)	2.86	2.01	4.29	2.15
Cu (g/kg)	0.86	0.06	19.29	0.06
Zn (g/kg)	0.02	0.13	0.08	0.13
Mn (mg/kg)	4.88	2.48	54.44	0.00
K (g/kg)	3.52	8.44	8.34	8.68
Mg (g/kg)	1.16	0.59	2.28	0.49
Co (mg/kg)	0.09	0.05	0.57	0.04
Se (mg/kg)	0.03	0.15	0.43	0.01
S (g/kg)	0.01	3.98	0.01	2.31
Fe (mg/kg)	32.80	16.70	452.35	1.46

Note: The nutritional level of the control group was calculated based on the highest daily supplementary feeding amount of 0.375 kg DM in late gestation. The nutritional contents of corn and corn straw refer to the Nutrient Requirements of Cashmere Goats, and the nutritional contents of soybean meal refer to the 31st edition of Chinese feed composition and nutritional value table.

**Table 3:** Nutrition surplus and deficit of cashmere goats during gestation under grazing conditions (dry matter basis).

Item	Early Gestation			Late Gestation		
NL	NL-UR	NL-SR	UR-SR	NL-UR	NL-SR	UR-SR
DMI (kg/d)	0.85	1.38	−0.53	0.67	1.60	−0.93
DE (MJ/d)	6.45	13.51	−7.06	6.28	15.57	−9.29
CP (g/d)	52.56	119.80	−67.24	25.99	142.72	−116.73
Ca (g/d)	4.69	3.75	0.94	2.04	4.62	−2.58
P (g/d)	0.60	2.69	−2.09	0.28	2.97	−2.69
Cu (mg/d)	2.89	38.03	−35.14	2.20	43.82	−41.62
Fe (mg/d)	853.46	55.39	798.07	1007.30	63.83	943.47
Zn (mg/d)	10.79	87.24	−76.45	7.78	100.53	−92.75
Mn (mg/d)	49.21	25.18	24.03	29.96	25.74	4.22
K (mg/d)	1651.63	6626.28	−4974.65	1150.81	7354.2	−6203.39
Mg (mg/d)	846.26	879.18	−32.92	827.27	916.45	−89.18
Co (mg/d)	0.83	0.15	0.68	0.44	0.18	0.26
Se (mg/d)	0.13	0.22	−0.09	0.14	0.23	−0.09
S (mg/d)	5.72	2354.07	−2339.35	1060.07	2712.79	−1652.72

Note: NL is nutrition level, NL-UR is goats’ nutrition level under grazing, NL-SR is goats’ nutrition level that the standard recommended and UR-SR is the gap between nutrient level under grazing and the corresponding feeding standard.

**Table 4:** Effect of supplementary feeding on cashmere production performance.

Item	Control Group	Supplemented Group	*p*-Value
Cashmere length before supplementation (cm)	7.35 ± 0.62	7.78 ± 0.74	0.42
Cashmere length after supplementation (cm)	8.47 ± 0.68	9.19 ± 0.57	0.76
Cashmere length growth volume (cm)	1.12 ± 0.53	1.41 ± 0.63	0.37
Cashmere fineness before supplementation (µm)	15.22 ± 0.59	14.75 ± 0.68	0.73
Cashmere fineness after supplementation (µm)	15.52 ± 0.54	14.88 ± 0.77	0.16
Cashmere yield (g)	715.36 ± 132.38	771.80 ± 153.46	<0.01

**Table 5:** Effect of supplementary feeding on weight after shearing of goats (kg).

Item	Control Group	Supplemented Group	*p*-Value
Average body weight after shearing	36.09 ± 4.32	38.15 ± 4.31	<0.01

**Table 6:** Effect of supplementary feeding on kid production.

Item	Control Group	Supplemented Group
Number of breeding goat	278	278
Number of goats producing kids	245	248
Total number of kids	280	278
Number of goats with single-born kid	210	219
Number of goats with twin-birth kid	35	28
Kidding rate (%)	114.29	112.10
Single birth rate (%)	85.71	88.31
Twinning rate (%)	14.29	11.29

**Table 7:** Effect of supplementary feeding on newborn weight of kids (kg).

Item	Control Group	Supplemented Group	*p*-Value
Newborn weight of kid	2.76 ± 0.27	2.87 ± 0.28	<0.01
Single-born kid weight	2.80 ± 0.24	2.92 ± 0.27	<0.01
Twin-birth kid weight	2.63 ± 0.25	2.64 ± 0.13	0.83

**Table 8:** Effect of supplementary feeding on hair follicle development.

Item	Control Group	Supplemented Group	*p*-Value
PF density (n/mm^2^)	8.12 ± 0.34	8.23 ± 0.28	0.82
SF density (n/mm^2^)	56.76 ± 2.31	59.91 ± 0.467	0.17
Mature SF density (n/mm^2^)	30.63 ± 1.84	34.37 ± 1.89	0.01
S:P	7.71 ± 0.35	8.13 ± 0.20	0.33

## Data Availability

Publicly available datasets were analyzed in this study, and these have been referenced in the manuscript.

## Supplementary Materials

The following supporting information can be downloaded at: https://www.mdpi.com/article/10.3390/ani13030473/s1, Table S1: Concentrations of alkanes of forage, feces and corn during gestation (mg/kg DM); Table S2: Forage nutrient levels during gestation (dry matter basis); Table S3: Contents of mineral elements of eating grass during pregnancy (mg/kg DM).
