# Supplementary material for: Supplementary Feeding of Grazing Inner Mongolian Cashmere Goats during Pregnancy—Based on “Nutrient Requirements of Cashmere Goats”
Source: Animals (Basel). 2023 Jan 29;13(3):473. doi: 10.3390/ani13030473 (PMC9913870; doi:10.3390/ani13030473)
Supplement: Supplementary file 1 [file animals-13-00473-s001.zip › animals-2131455-supplementary.pdf]

animals MDPI

Supplementary Table S1. Concentrations of alkanes of forage, feces and corn during gestation (mg/kg DM).

Item	Sample	C_{25}	C_{27}	C_{29}	C_{31}	C_{32}	C_{33}	C_{35}
Early gestation	*Stipa breviflora Griseb*	76.92	334.44	1435.39	1471.47	0.00	245.13	10.57
	Peganum harmala L	8.92	18.64	0.00	22.25	0.00	2.49	0.00
	Oxytropis aciphylla Ledeb	17.47	28.09	32.14	67.71	0.00	3.75	0.00
	Corn	0.00	0.00	0.00	0.06	0.00	0.43	0.00
	Faecal sample 1	41.71	266.40	1176.14	1280.15	49.02	214.39	13.15
	Faecal sample 2	47.06	320.49	1379.42	1434.64	113.94	299.58	19.27
	Faecal sample 3	54.92	337.43	1421.70	1507.17	42.80	236.83	14.10
	Faecal sample 4	47.09	307.64	1336.83	1492.47	64.49	245.41	16.74
	Faecal sample 5	45.70	304.05	1270.30	1399.67	43.86	259.54	18.89
	Faecal sample 6	50.33	316.94	1353.85	1479.41	43.41	276.04	16.44
	Faecal sample 7	64.64	383.04	1624.26	1604.81	151.09	246.77	16.00
	Faecal sample 8	66.90	373.81	1491.92	1376.10	55.17	232.52	17.76
Late gestation	*Stipa breviflora Griseb*	65.31	254.89	550.08	942.82	17.97	163.83	9.08
	Peganum harmala L	10.82	18.54	58.86	11.65	0.00	0.00	0.00
	Oxytropis aciphylla Ledeb	23.24	21.72	85.00	40.43	0.00	4.02	0.00
	Faecal sample 1	59.46	332.90	659.49	1094.62	131.08	192.54	15.54
	Faecal sample 2	36.03	197.92	444.98	831.84	99.02	150.88	12.31
	Faecal sample 3	43.71	247.61	557.12	1064.26	102.19	193.07	13.94
	Faecal sample 4	51.79	284.15	650.26	1265.07	116.06	235.81	15.32
	Faecal sample 5	51.44	284.73	574.04	967.23	112.21	178.77	14.57
	Faecal sample 6	52.30	317.31	611.73	976.55	96.78	164.02	14.78
	Faecal sample 7	47.44	269.16	545.05	903.43	84.94	161.72	13.40
	Faecal sample 8	52.73	287.19	606.87	1075.55	85.74	197.07	15.42

Supplementary Table S2. Forage nutrient levels during gestation (dry matter basis).

Species	Stage	DM (%)	CP (%)	Ca (%)	P (%)	NDF (%)	ADF (%)	GE (MJ/kg)
Stipa breviflora Griseb	early gestation	96.10	6.17	0.55	0.07	59.68	39.01	18.93
	late gestation	97.18	4.46	0.36	0.03	66.30	53.71	19.53
Peganum harmala L	early gestation	95.52	11.71	3.71	0.10	31.75	27.59	15.69
	late gestation	96.93	10.92	2.09	0.06	36.46	32.33	15.76
Oxytropis aciphylla Ledeb	early gestation	96.01	8.14	2.43	0.06	49.16	42.37	17.37
	late gestation	97.03	1.81	0.11	0.09	57.14	50.91	17.48

Supplementary Table S3. Contents of mineral elements of eating grass during pregnancy (mg/kg DM).

Species	Stage	Cu	Fe	Zn	Mn	K	Mg	Co	Se	S
Stipa breviflora Griseb	early gestation	3.39	1001.91	12.67	57.77	1938.90	993.45	0.98	0.16	6.72
	late gestation	2.51	697.04	8.80	51.58	1160.84	834.49	0.63	0.16	1492.11
Oxytropis aciphylla Ledeb	late gestation	5.77	4158.87	20.67	20.37	3530.54	2537.97	2.14	0.35	1836.06
